# Characterization of Innate Responses Induced by PLGA Encapsulated- and Soluble TLR Ligands *In Vitro* and *In Vivo* in Chickens

**DOI:** 10.1371/journal.pone.0169154

**Published:** 2017-01-03

**Authors:** Tamiru N. Alkie, Khaled Taha-Abdelaziz, Neda Barjesteh, Jegarubee Bavananthasivam, Douglas C. Hodgins, Shayan Sharif

**Affiliations:** 1 Department of Pathobiology, University of Guelph, Guelph, Ontario, Canada; 2 Pathology Department, Beni-Suef University, Al Shamlah, Beni-Suef, Egypt; Universidad de Castilla-La Mancha, SPAIN

## Abstract

Natural or synthetic Toll-like receptor (TLR) ligands trigger innate responses by interacting with distinct TLRs. TLR ligands can thus serve as vaccine adjuvants or stand-alone antimicrobial agents. One of the limitations of TLR ligands for clinical application is their short half-life and rapid clearance from the body. In the current study, encapsulation of selected TLR ligands in biodegradable poly(D,L-lactide-co-glycolide) polymer nanoparticles (PLGA NPs) was examined *in vitro* and *in vivo* as a means to prolong innate responses. MQ-NCSU cells (a chicken macrophage cell line) were treated with encapsulated or soluble forms of TLR ligands and the resulting innate responses were evaluated. In most cases, encapsulated forms of TLR ligands (CpG ODN 2007, lipopolysaccharide and Pam3CSK4) induced comparable or higher levels of nitric oxide and cytokine gene expression in macrophages, compared to the soluble forms. Encapsulated CpG ODN, in particular the higher dose, induced significantly higher expression of interferon (IFN)-γ and IFN-β until at least 18 hr post-treatment. Cytokine expression by splenocytes was also examined in chickens receiving encapsulated or soluble forms of lipopolysaccharide (a potent inflammatory cytokine inducer in chickens) by intramuscular injection. Encapsulated LPS induced more sustained innate responses characterized by higher expression of IFN-γ and IL-1β until up to 96 hr. The ability of TLR ligands encapsulated in polymeric nanoparticles to maintain prolonged innate responses indicates that this controlled-release system can extend the use of TLR ligands as vaccine adjuvants or as stand-alone prophylactic agents against pathogens.

## Introduction

Cells of the innate system such as macrophages and dendritic cells rely on pattern recognition receptors (PRRs) to detect pathogen-associated molecular patterns (PAMPs) [1]. Toll-like receptors (TLRs) are the most widely studied class of PRRs [2], which recognize different classes of natural TLR ligands representing microbial structural components [2,3] and synthetic PAMP mimics [4]. The recognition of TLR ligands by TLRs triggers intracellular signaling pathways such as myeloid differentiation factor 88/TRIF (TIR-domain-containing adapter-inducing interferon-β [5,6] and expression of key regulatory factors such as interferon regulatory factors [4,7] for induction of effector molecules including cytokines [8]. In avian species, the basic biology and downstream signaling pathways initiated as a result of TLR-PAMP interactions are, to some extent, similar to those in mammals [9,10], although some differences exist between avian and mammalian molecules and pathways.

Modulation of innate responses by TLR ligands such as CpG oligodeoxynucleotides (ODN), recognized by TLR9 in mammals [11] and TLR21 in chickens [12] and lipopolysaccharide (LPS), recognized by TLR4 [3] has profound effects on generation of adaptive immune responses [13]. As vaccine adjuvants, TLR ligands enhance the proliferation and antigen presentation capabilities of innate immune system cells, a desired quality for vaccines [14]. Potent antimicrobial effects of TLR ligands have been reported for vertebrate species including chickens [15]. Nevertheless, key challenges remain for the use of TLR ligands as vaccine adjuvants or stand-alone antimicrobial compounds. Some of these challenges include the short half-life and rapid clearance of TLR ligands [16] as well as the potential to induce uncontrolled systemic innate responses which may have deleterious effects on the host [17]. An alternative slow controlled-release method for TLR ligands could maintain a desired innate response and circumvent the need for repeated administration. Encapsulation or adsorption of these ligands in polymeric microparticles or nanoparticles provides a slow controlled-release system [18], which would reduce *in vivo* degradation of TLR ligands and increase their potency. Poly(D,L-lactide-co-glycolide) (PLGA) is a biodegradable polymer approved by the Food and Drug Administration in the USA and by the European Medicine Agency for delivery of pharmaceuticals in humans [19–21]. PLGA has been used to deliver antigens and adjuvants in veterinary species such as chickens [22]. PLGA particulates in the form of microparticles or nanoparticles are flexible and tunable, their surfaces can be modified [23] and ligands can be adsorbed to the surface [22] or encapsulated in their interior matrix [24] for induction of potent and long lasting immune responses. In this regard, encapsulation of CpG ODN or LPS mimetics in PLGA microparticles (PLGA MPs) enhances the adjuvant activity of these ligands [24].

Stimulation of chicken TLRs with their respective ligands induces an array of cytokines and other innate molecules, but there is limited information available on how these responses are modulated when ligands are delivered with PLGA as a particulate carrier. The present study was conceived to determine the ability of TLR ligands encapsulated in PLGA nanoparticles (PLGA NPs) to trigger and sustain innate responses in chickens.

## Materials and Methods

### Chemicals

Poly(D,L-lactide-co-glycolide) (Resomer^®^ RG 503H, free carboxylic acid, MW 24–38 kD), dichloromethane, polyvinyl alcohol (PVA, MW 30–70 kD, 87–90% hydrolyzed), polyethylenimine (linear, MW 2.5 kD), poloxamer 407, class B CpG ODN 2007 (phosphorothioate backbone modified with a sequence of 5'-TCGTCGTTGTCGTTTTGTCGTT-3' and its non-CpG ODN form, FITC-LPS (derived from *Escherichia coli* O111:B4) and lipopolysaccharide (LPS) (derived from *Escherichia coli* O111:B4, TLR ligand tested) were obtained from Sigma-Aldrich (Oakville, ON, Canada). Pam3CSK4 and Rhodamine-Pam3CSK4 (endotoxin level tested) were obtained from InvivoGen (San Diego, CA, USA). Quant-iT™ OliGreen^®^ ssDNA reagent was from Life Technologies.

### Preparation and characterization of PLGA NPs

Synthetic oligodeoxynucleotides (here ODNs refer to CpG ODN or non-CpG ODN) were encapsulated in PLGA NPs as described [25,26], with modifications made in the volume of primary emulsions. LPS or Pam3CSK4 were encapsulated as described [24]. The encapsulation efficiency of CpG ODN or non-CpG ODN was increased by forming a complex of each of the ODNs with polyethylenimine at a molar ratio of 5 (primary amino in polyethylenimine to phosphate in ODNs) calculated according to previous work [27]. Briefly, 434 μg of CpG ODN or non-CpG ODN in 125 μL nuclease free water was added to 125 μL polyethylenimine (0.29 mg dissolved in 150 mM NaCl) and were mixed at 700 rpm for 20 min. The resulting complex (250 μL) was sonicated with 1250 μL cold 4.5% PLGA (PLGA dissolved in dichloromethane) for 1 minute (10 sec sonication followed by 5 sec pause at 40% amplitude) (Ultrasonic processor, 3 mm probe diameter, Fisher Scientific, Ottawa, ON, Canada). The primary emulsion for LPS (2.4 mg/250 μL water) and Pam3CSK4 (1 mg/500 μL water) was generated by sonicating in 1250 μL and 1000 μL cold 4.5% PLGA, respectively. The primary emulsion of each of the TLR ligands (1500 μL) was further sonicated for 2 min (10 sec sonication and 5 sec pause at 60% amplitude) in 3250 μL cold 2% PVA/1% poloxamer. The resulting emulsion was poured into 50 mL 2% PVA/1% poloxamer solution and stirred with a magnetic stirrer at room temperature to allow dichloromethane evaporation. PLGA NPs were pelleted at 25,000 g for 30 min at 4°C, washed five times in water (HyClone^®^ HyPure, Thermo Scientific), resuspended and snap frozen for lyophilization (FreeZone^®^18 Liter Freeze Dry System, Labconco, Kansas City, MO, USA). Supernatant from each washing step was tested in cell culture to detect traces of non-encapsulated ligands. Blank PLGA NPs (mock PLGA NPs) were produced similarly. Lyophilized PLGA NPs were sterilized by γ-irradiation with a Co^60^-derived dose of 2.5 MRad using the Gamma Cell 220 type B (U) irradiation facility of the Southern Ontario Centre for Atmospheric Aerosol Research (University of Toronto, Toronto, Canada). Irradiated PLGA NPs were stored at 4°C until use.

The size and zeta potential (surface charge) of PLGA NPs were determined by dynamic light scattering (Zetasizer Nano, Malvern Instruments, Worcestershire, UK). The encapsulation efficiency of CpG ODN or non-CpG ODN was determined by dissolving lyophilized PLGA NPs encapsulating CpG ODN or non-CpG ODN (1 mg/mL in TE buffer) in 1 mL dichloromethane and shaking for 1 hr at room temperature [28]. The quantity of CpG ODN or non-CpG ODN released into the aqueous phase was measured using the Quant-iT™ OliGreen^®^ ssDNA reagent and kit system (Invitrogen) and a GloMax^®^-Multi Detection System-Fluorometer (Promega, Madison, WI). The encapsulation efficiency of LPS and Pam3CSK4 was determined by encapsulating FITC-LPS or Rhodamine-Pam3CSK4 in PLGA NPs. The fluorescence intensity of FITC and Rhodamine extracted from a determined quantity of PLGA NPs with hot phenol-water [29] was measured using a GloMax^®^-Multi Detection System-Fluorometer.

### Assessment of the stimulatory property of encapsulated- and soluble TLR ligands in chicken macrophages

MQ-NCSU cells, a chicken macrophage cell line were seeded (2x10^6^ cells/mL) in 12-well Corning Costar cell culture plates (Sigma-Aldrich) and incubated for 8 hr at 40°C and 5% CO_2_ in a humidified incubator in complete McCoy’s 5A (Modified) and L-15 Leibovitz medium (Invitrogen). The cell monolayer was washed twice with Dulbecco’s Modified Eagle's Medium (DMEM) (Gibco, NY, USA) supplemented with 10% heat-inactivated fetal calf serum, 1% penicillin-streptomycin and 25 mM HEPES and treated with encapsulated- and soluble TLR ligands in DMEM for 24 hr. Cell activation was assessed by measuring nitrite in cell culture supernatants using the Griess Reagent System (Promega, Madison, WI). The chicken macrophage cell line used in this study responds well to different classes and doses of TLR ligands selected for the current study [30].

Two different doses of the following TLR ligands (encapsulated and soluble forms) were used to treat MQ-NSCU cells; each dose consisting of six replicates: LPS (0.1 μg/mL and 1 μg/mL), CpG ODN (1 μg/mL and 5 μg/mL), and Pam3CSK4 (1 μg/mL and 5 μg/mL) and non-CpG ODN (5 μg/mL). The doses for the current experiment were selected based on previous works [30,31]. The control groups received DMEM or mock PLGA NPs. To prevent aggregation of PLGA NPs in DMEM [32] and increase PLGA NPs-cell interactions, plates were centrifuged at 150 g for 5 min. After stimulation for 3, 12 or 18 hr, total RNA was extracted from the cells using TRIzol^®^ Reagent (Invitrogen). RNA was treated with DNAse (DNA Free^®^, Ambion, Austin, TX) to remove contaminating DNA. Complementary single-stranded DNA *(*cDNA) was synthesized from 1 μg of purified RNA using oligo(dT)_20_ primers and Superscript^®^ II First Strand Synthesis System (Invitrogen).

### *In vivo* assessment of encapsulated- and soluble TLR ligands

One-day-old female broiler chickens (n = 127) were obtained from Stratford Chick Hatchery (Stratford, ON, Canada). At the age of fourteen-days, they were randomly assigned to 6 groups (n = 20-25/group). Groups 1 and 2 were treated intramuscularly with low (25 μg/bird) and high (100 μg/bird) doses of soluble LPS, respectively. Chickens in group 3 received PLGA NPs encapsulating 25 μg LPS/bird and chickens in group 4 received PLGA NPs encapsulating 100 μg LPS/bird. Groups 5 and 6 were treated with mock PLGA NPs and phosphate buffered saline (PBS), respectively. All injections were prepared in 200 μL PBS and administered in the thigh muscles. During the entire experiments, our experimental animals were maintained in the animal isolation facility of the Ontario Veterinary College, University of Guelph. All animal experiments were approved by the University of Guelph Animal Care Committee. Accordingly, chickens were kept in groups in isolators that were enriched and supplied with bedding. Feed and water were supplied *ad libitum*. Chickens in each group were administered with the appropriate dose of compounds as indicated above and were monitored every day for clinical signs and mortality and feed and water intake. Weight gain was monitored and liver, kidneys and muscles (at the injection sites) were examined at necropsy. During the experimental periods, chickens did not show any signs of morbidity and remained healthy. Moreover, there were no mortality, and lesions were not present in all organs examined. At the end of the experimental period chickens were euthanized humanely using carbon dioxide inhalation.

At 3, 18, 48 or 96 hr post-treatment, 5–6 chickens per time point were euthanized and their spleens were collected. RNA was extracted from splenocytes and cDNA synthesis was conducted as described elsewhere in this paper.

### Real-time PCR

Expression of selected cytokine genes was quantified by SYBR Green real-time PCR using the LightCycler^®^ 480 II system (Roche Diagnostics GmbH, Mannheim, Germany). The primers and PCR conditions are indicated in Table 1. Gene expression levels were normalized to chicken β-actin and fold changes were generated with REST (REST software version 2009, Qiagen).

**Table 1:** Genes and primer sequences used for real-time PCR.

Target genes	Primer sequences	References
β-actin	F: 5'-CAACACAGTGCTGTCTGGTGGTA-3'	[33]
	R: 5'-ATCGTACTCCTGCTTGCTGATCC-3'	
IFN-β	F: 5'-GCCTCCAGCTCCTTCAGAATAC G-3'	[34]
	R: 5'-CTGGATCTGGTTGAGGAGGCTGT-3'	
IFN-α	F: 5'-ATCCTGCTGCTCACGCTCCTTCT-3'	[33]
	F: 5'-GGTGTTGCTGGTGTCCAGGATG-3'	
IFN-γ	F: 5'-ACACTGACAAGTCAAAGCCGCACA-3'	[35]
	R: 5'-AGTCGTTCATCGGGAGCTTGGC-3'	
IL-1β	F: 5'-GTGAGGCTCAACATTGCGCTGTA-3'	[36]
	R: 5'-TGTCCAGGCGGTAGAAGATGAAG-3'	
IL-8	F: 5'-CCAAGCACACCTCTCTTCCA-3'	[36]
	R: 5'-GCAAGGTAGGACGCTGGTAA-3'	

### Statistics

Data obtained from LPS and Pam3CSK4 were log transformed and analyzed by Proc GLM (SAS 9.3, Cary, NC). The Kruskal-Wallis (a nonparametric) test was used to analyze data obtained from CpG ODN and for *in vivo* experiment as the data in these studies were not normally distributed. Macrophages treated with non-CpG ODN in either forms, or with mock PLGA NPs did not up-regulate cytokine gene expression to significant levels. Non-CpG ODN and mock PLGA NPs, therefore, served as qualitative controls and were not considered further in the analyses. Data are given as the mean fold change (± standard error of the mean) of the relative gene expression in the TLR ligand treated groups (encapsulated or soluble forms) compared to the control (culture medium for *in vitro* and phosphate buffered saline for the *in vivo* experiment) groups. A value of *P* <0.05 was considered significant.

## Results

### Characterization of PLGA NPs

TLR ligands were encapsulated in PLGA NPs and characterized for their physical properties and sterilized with γ-irradiation for *in vitro* and *in vivo* studies. The particles had comparably similar sizes with low polydispersity indices that measure the width of size distributions of the particles. The particles had varying surface charges and encapsulation efficiency (Table 2). PLGA NPs encapsulating LPS and Pam3CSK4 had lower zeta values due to additional surface adsorption of these TLR ligands during the encapsulation processes. The lower zeta value of PLGA NPs encapsulating CpG ODN or non-CpG ODN compared to mock PLGA NPs was expected as traces of polyethylenimine, used to form complexes with CpG ODN or non-CpG ODN were incorporated on the surface of the particles.

**Table 2:** Physical properties of PLGA NPs encapsulating different classes of TLR ligands.

PLGA NPs formulation	Diameter (nm)	Zeta potential (mV)	Encapsulation efficiency (%)
mock PLGA NPs	624±4	-33 ± 3	NA
PLGA ODN NPs	642±10	-5± 1	74±1
PLGA LPS NPs	673±15	-2±2	72±1
PLGA Pam3CSK4 NPs	667±25	0.1±0.1	84±2

Three measurements were done at 25°C. NA indicates not applicable.

### Nitric oxide production by macrophages

**Fig 1:**
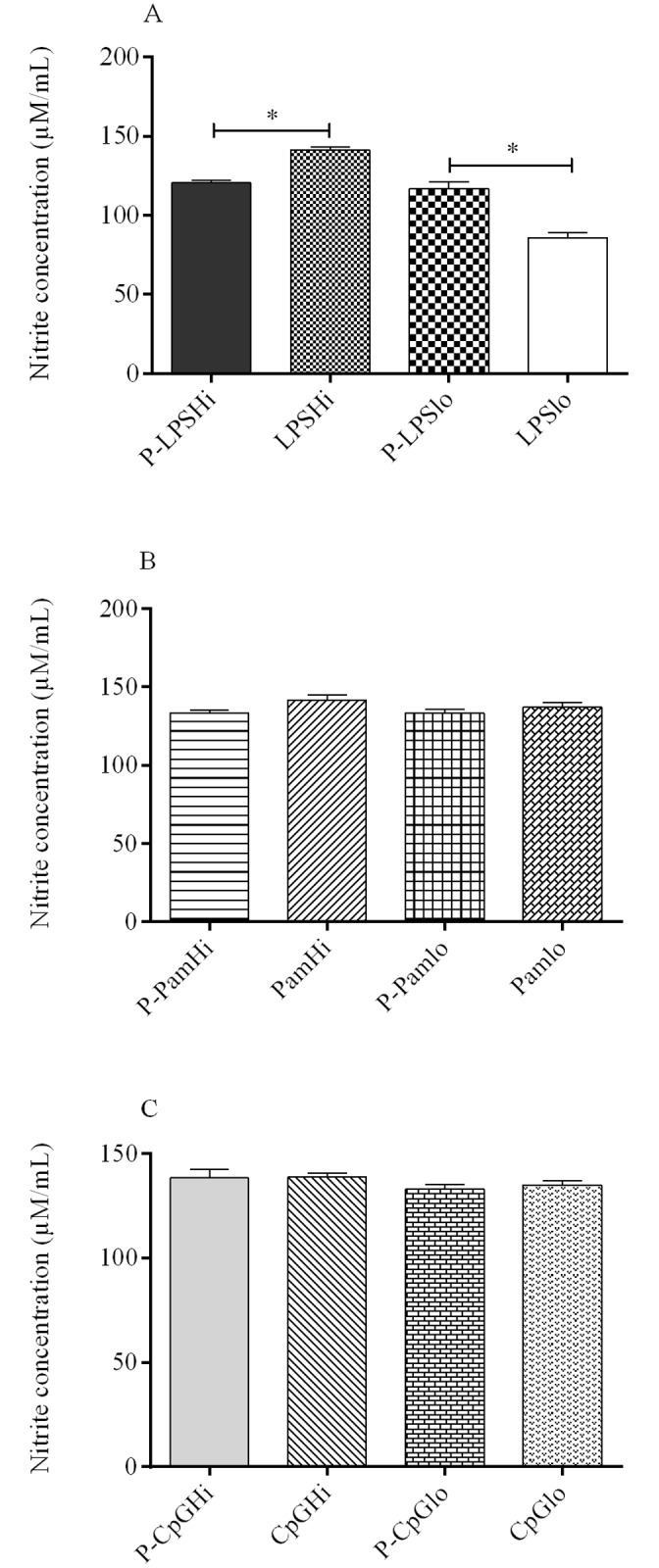
Nitric oxide (NO) production from chicken macrophages (six replicates/group) stimulated with encapsulated and soluble forms of three TLR ligands, measured by Griess reagent system. (A). LPS; (B). Pam3CSK4, and (C). CpG ODN. Significance (*P* <0.05) between delivery systems (encapsulated and soluble TLR ligands) within a treatment dose was determined. P-LPSHi—encapsulated high dose LPS; P-LPSlo—encapsulated low dose LPS; LPSHi—soluble high dose LPS; LPSlo—soluble low dose LPS. P-PamHi—encapsulated high dose Pam3CSK4; P-Pamlo—encapsulated low dose Pam3CSK4; PamHi—soluble high dose Pam3CSK4; Pamlo—soluble low dose Pam3CSK4. Similar designations were made for CpG ODN. * indicates significant difference.

Macrophages produced nitric oxide as early as 24 hr after stimulation with encapsulated and soluble TLR ligands consistent with macrophage activation by PLGA NPs (S1 Table). PLGA LPS low (PLGA encapsulated low dose LPS, designated as P-LPSlo) induced significantly higher (p = 0.02) amounts of nitric oxide compared to low dose soluble LPS (LPSlo). In contrast, PLGA LPS high (PLGA encapsulated high dose LPS, designated P-LPSHi) induced significantly lower amounts of nitric oxide compared to the same dose of soluble LPS (LPSHi) (Fig 1A). When delivered as encapsulated forms, Pam3CSK4 (Fig 1B) and CpG ODN (Fig 1C) induced comparable nitric oxide production relative to their soluble forms.

### Evaluation of interferon (IFN) -γ and IFN-β expression in chicken macrophages

**Fig 2:**
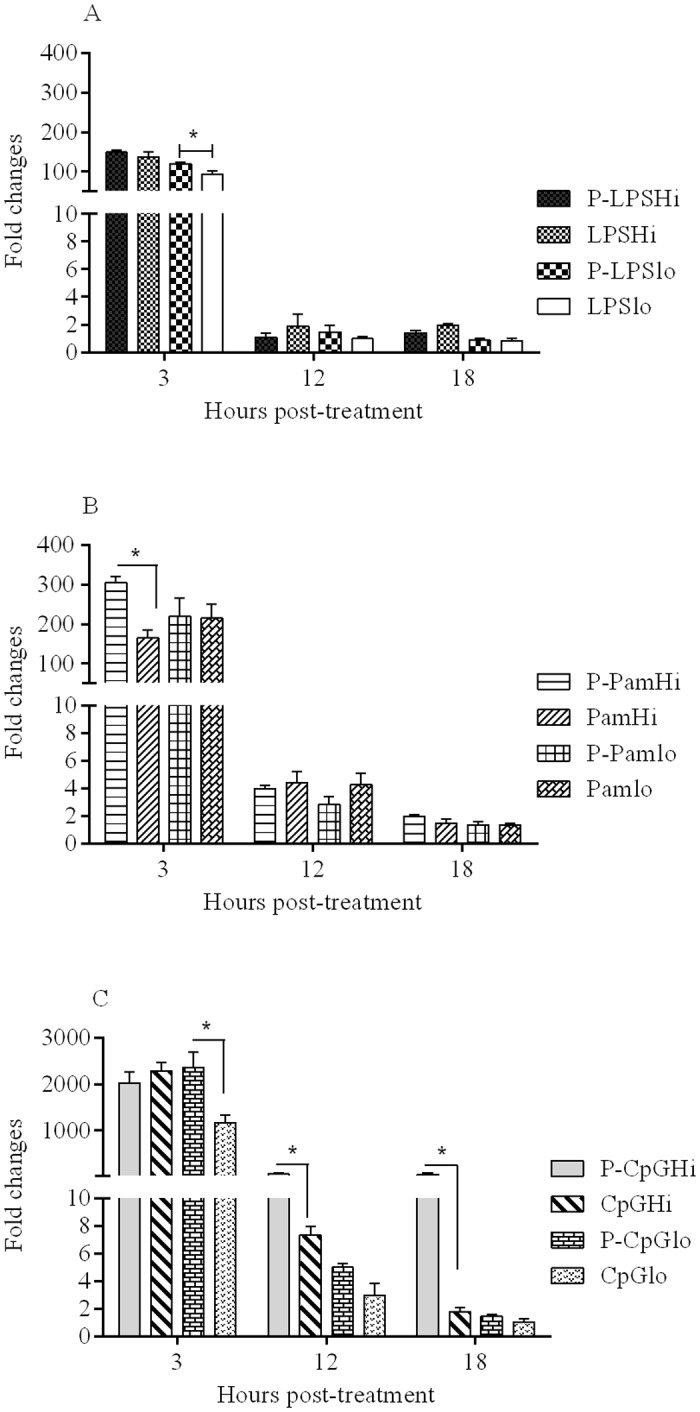
Expression profiles of IFN-γ for (A). LPS; (B). Pam3CSK4, and (C). CpG ODN. Chicken macrophages (six replicates/group) were treated with encapsulated and soluble forms of the three TLR ligands and mean fold expression of IFN-γ in treated groups were compared to the cell culture medium treated group. Significance (*P* <0.05) was tested between delivery systems (encapsulated and soluble TLR ligands) within a dose and at a given time point. * indicates significant difference.

The encapsulated form of low dose LPS (P-LPSlo) resulted in significantly higher (p = 0.01) expression of IFN-γ (120 mean fold compared to medium control), whereas the soluble low dose LPS (LPSlo) resulted in 94 fold increase (mean fold change compared to medium control) at 3 hr post-treatment (Fig 2A). The higher dose of LPS (encapsulated or soluble) did elicit higher levels of IFN-γ expression at 3 hr post-treatment; however, this response did not differ between the two forms (P-LPSHi versus LPSHi). At 12 and 18 hr, the expression of IFN-γ was not significant for soluble and encapsulated LPS at any doses (Fig 2A).

The encapsulated Pam3CSK4 high dose (P-PamHi) resulted in significantly higher (p = 0.02) IFN-γ expression (300 mean fold increase compared to the expression of IFN-γ in medium treated group) at 3 hr post-treatment, whereas the same dose of Pam3CSK4 in a soluble form (PamHi) resulted in 160 mean fold increase (Fig 2B). The expression of IFN-γ did not differ when Pam3CSK4 was used as low dosage formulation (P-Pamlo versus Pamlo). At 12 and 18 hr, the expression of IFN-γ was not significant for soluble and encapsulated Pam3CSK4 at both dosage formulations.

Encapsulated LPS or Pam3CSK4 and their soluble forms did not upregulate the expression of IFN-α and IFN-β transcripts in chicken macrophages (S2 Table).

**Fig 3:**
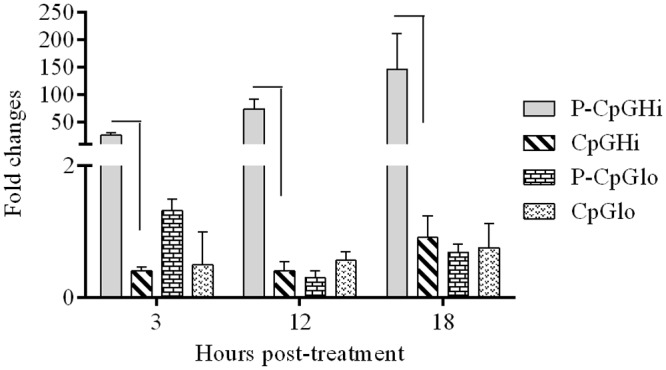
Expression level of IFN-β by stimulation with CpG ODN. Chicken macrophages (six replicates/group) were treated with encapsulated and soluble CpG ODN. Mean fold expression of IFN-β in treated groups were compared to the cell culture medium treated group. Significance (*P* <0.05) was tested between delivery systems within a dose and at a given time point. * indicates significant difference.

For CpG ODN, irrespective of the doses and forms of delivery, chicken macrophages expressed significant amounts of IFN-γ transcript at 3 hr post-treatment (Fig 2C). The high dose of encapsulated CpG ODN (P-CpGHi) caused significantly higher (p < 0.0001) expression of IFN-γ at 12 and 18 hr (60 mean fold compared to medium group; Fig 2C); however, its soluble counterpart had caused 2–7 mean fold increase. Moreover, a high dose of encapsulated CpG ODN (P-CpGHi) resulted in significantly higher (p < 0.0001) IFN-β expression at 3 (27 mean fold), 12 (74 mean fold) and 18 hr (146 mean fold compared to medium group) post-treatment compared to its soluble form (Fig 3). The low dose of CpG ODN did not induce IFN-β expression in both formulation types.

### Evaluation of interleukin (IL)-1β expression in chicken macrophages

**Fig 4:**
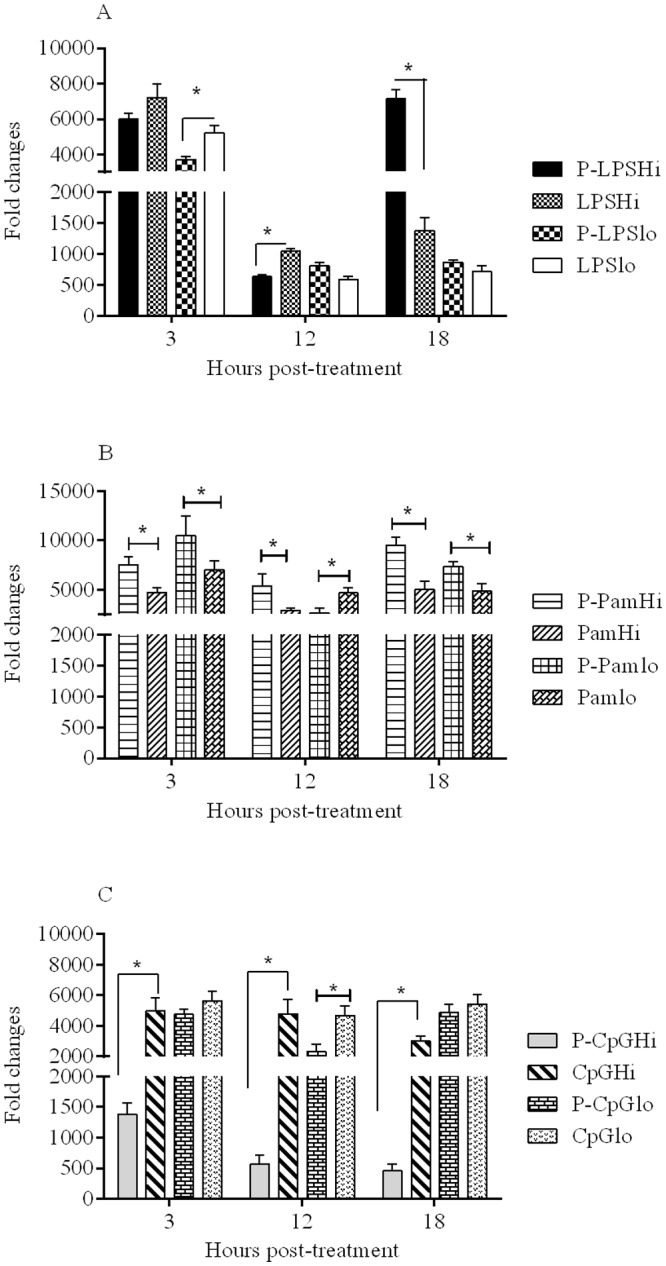
IL-1β mRNA expression in chicken macrophages stimulated with (A). LPS; (B). Pam3CSK4; and (C) CpG ODN. Chicken macrophages (six replicates/group) were treated with encapsulated and soluble forms of the three TLR ligands and mean fold expression of IL-1β in treated groups were compared to the cell culture medium treated group. Significance (*P* <0.05) was tested between delivery systems within a dose and at a given time point. * indicates significant difference.

At all time points, all TLR ligands caused elevated levels of IL-1β expression in macrophages compared to untreated and mock PLGA NP groups. At 3 hr post-treatment, the soluble forms of LPS, particularly the low dose formulation showed significant upregulation of IL-1β compared to the encapsulated form. At 18 hr post-treatment (Fig 4A), PLGA LPS high dose (P-LPSHi) induced significantly higher (p < 0.0001) expression of IL-1β (7,000 mean fold compared to medium) compared to the same dose of soluble LPS (1,400 mean fold higher than the medium treated group).

For Pam3CSK4, except at 12 hr post-treatment for the low dose, the encapsulated forms resulted in significantly higher (p < 0.05) levels of IL-1β expression compared to the soluble forms (Fig 4B).

For CpG ODN, in contrast to the effects seen on expression of IFN-γ and IFN-β, the soluble form of CpG ODN at high dose resulted in significantly higher (p < 0.0001) expression of IL-1β than the encapsulated form at all time points investigated (Fig 4C). Treatment with CpG ODN at low dose (Fig 4C), was associated with high expression of IL-1β (2,000 mean fold higher than medium control) at all time points, regardless of delivery system.

### Expression of cytokine genes in spleen

A four-day time course study was conducted to determine the kinetics of innate responses in the spleens of chickens treated intramuscularly with encapsulated or soluble LPS. Chickens injected with encapsulated and soluble LPS had very similar weight gains over a period of one week compared to control (mock PLGA NP) group (S3 Table) and did not show any clinical signs of disease, or lesions upon necropsy.

**Fig 5:**
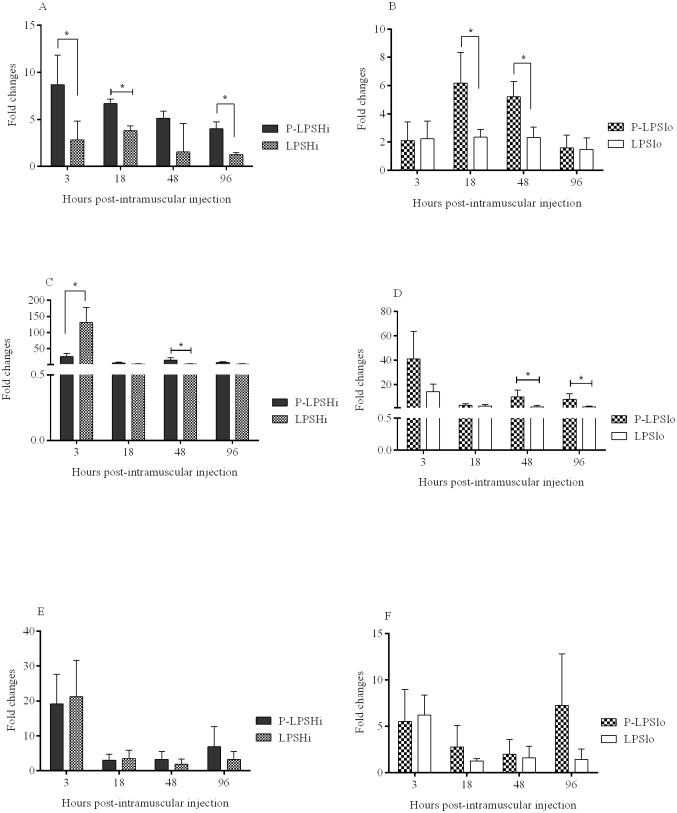
Quantification of IFN-γ (A, B), IL-1β (C, D) and IL-8 (E, F) expression in spleen of chickens intramuscularly injected with encapsulated and soluble LPS. At 3, 18, 48 and 96 hr post-injection, spleens were collected and the relative expression of cytokine genes was determined. Bars represent mean fold expression of cytokines in treated chickens compared to the control chickens. Cytokine gene expressions were compared between delivery systems (encapsulated and soluble) within a dose and at a given time point. * indicates significant difference at *P* <0.05.

Differences in gene expression patterns in spleen were observed between groups of chickens receiving encapsulated and soluble LPS (S4 Table). For encapsulated high dose LPS (P-LPSHi), IFN-γ expression was significantly higher (p < 0.05) at 3 (8 mean fold compared to PBS treated group), 18 (7 mean fold), and 96 hr (4 mean fold) post-treatment compared to the soluble counterpart (Fig 5A), whereas encapsulated low dose LPS (P-LPSlo) had significantly increased expression of IFN-γ at 18 (6 mean fold) and 48 hr (5 mean fold) (Fig 5B).

At 3 hr post-treatment expression of IL-1β was significantly higher (p < 0.05) for chickens receiving the high dose of soluble compared to high dose encapsulated LPS, but by 48 hr encapsulated LPS (high or low dose) caused higher expression of IL-1β (9–14 mean fold) (Fig 5C and 5D), compared to the soluble form. The low dose encapsulated LPS showed further upregulation of IL-1β at 96 hr (Fig 5D).

For IL-8, expression at 3 hr post-treatment was higher for chickens receiving encapsulated and soluble forms compared to that of control birds (Fig 5E and 5F), but was not different between the doses, in either form (Fig 5E and 5F). At 96 hr, a pattern was observed where the encapsulated forms resulted in higher IL-8 expression than the soluble forms.

## Discussion

PLGA NPs formulations have been tested as delivery systems for TLR ligands to improve the quantity, quality and duration of immune responses induced by vaccine antigens [18,20]. These ligands can be adsorbed to the surface of PLGA NPs [22], encapsulated [24] or chemically conjugated to particulate delivery systems [37] to boost adaptive immune responses by activating innate cells. Nonetheless, innate responses induced by surface adsorbed TLR ligands compared to responses induced by encapsulated forms are short lived, a characteristic which may limit the ability of these ligands to drive adaptive immune responses to higher levels [38]. The role of PLGA NPs as a slow, controlled-release system for TLR ligands in enhancing and sustaining innate responses is poorly understood. To this end, the potency of TLR ligands encapsulated in PLGA NPs to trigger and sustain higher innate responses was evaluated *in vitro* and *in vivo*. In this study, most PLGA NP formulated TLR ligands resulted in either similar or higher levels of cytokine gene expression and other molecules by macrophages compared to the soluble (non-encapsulated) forms, indicating that encapsulated ligands are immunologically active.

The current study demonstrated the capability of class B CpG ODN to enhance type I IFN expression like class A CpG ODNs [39], which are by nature stronger inducer of IFN-β. A higher level of IFN-γ as well as IFN-β expression induced by encapsulated class B CpG ODN is consistent with a previous report [28]. Other studies also revealed that CpG ODN adsorption to the surface of non-degradable polymers enhance innate responses *in vitro* in mononuclear phagocytic cells [26,40]. Such effects may be attributable to increased uptake of PLGA encapsulated CpG ODN by mononuclear phagocytic cells [28]. The induction of increased expression of type I IFNs from mononuclear phagocytic cells by class A CpG ODNs is due to the spontaneous formation of nanoparticle structures, which facilitate cellular uptake of class A CpG ODNs. In class A CpG ODNs, the self-assembly of the palindromic and poly-G sequences results in nanometer-sized multimers [41]. Pam3CSK4 contains highly positively charged amino acid moieties and showed higher cytokine-inducing ability when delivered as encapsulated form. This ligand also showed a strong tendency to adsorb to the surface of PLGA NPs during encapsulation and as such may improve particle uptake by directing the particles to TLR2. This may improve uptake and promote pro-inflammatory cytokine secretion [42]. TLR ligands, such as CpG ODN, induce a predominately of T helper 1 (Th1)-type response profile in vertebrate species. Therefore, a higher and long lasting Th1-polarized immune response can be induced by delivering class B CpG ODN encapsulated in polymeric particulate carrier systems [43].

A slow-release and prolonged immune stimulation profile *in vitro* for encapsulated TLR ligands was best demonstrated by a sustained increased expression of IFN-β by CpG ODN and IL-1β by LPS and Pam3CSK4 at the latest time points investigated. However, the expression of IFN-γ and IL-1β may be inversely related in this *in vitro* study. When IFN-γ was highly expressed, the expression of IL-1β was relatively lower for both soluble and encapsulated ligands. Recently, it has been shown that IFN-γ suppresses LPS induced IL-1β transcription by selectively attenuating the binding of NF-κB to the IL-1β promotor [44]. Delivery of TLR ligands by particulate carriers as in this *in vitro* study may reduce expression of pro-inflammatory cytokine genes compared to soluble ligands and thus limit inflammation.

The effects of encapsulation of LPS on induction of pro-inflammatory cytokines were further tested *in vivo* in chickens. In chickens, soluble LPS is potent inflammatory cytokine inducer. We noted two profiles of cytokine gene expression; immediate higher expression (as early as 3 hrs) and later, more sustained, responses characterized by an extended duration of expression of IFN-γ and IL-1β in spleen for up to 96 hr. In this study, encapsulation of LPS maintained a baseline intensity of inflammation, where the magnitude of cytokine expression at later time points did not exceed expression at early time points and hence was unlikely to induce harmful inflammatory responses.

The surface associated TLR ligands (inferred based on the surface charge of PLGA NPs) may lead to an immediate TLR activation and pro-inflammatory cytokine transcript expression in both *in vitro* and *in vivo* studies. TLRs on macrophages may recognize LPS and Pam3CSK4 presented on the surfaces of PLGA NPs. Recent evidence supports this observation as LPS adsorbed onto the surface of PLGA NPs induces an early onset of pro-inflammatory cytokine expression in dendritic cells [45,46]. The effects of surface adsorbed ligands were found to be similar to those of soluble TLR ligands for priming of innate cells to mount immediate cytokine responses [29,47], whereas the encapsulated portion of the TLR ligands sustain the response. Signaling in innate cells induced by priming with TLR ligands (where the signals involve MyD88/TRIF or NF-κB activating pathways), may precede NLRP3 inflammasome activation in macrophages and results in immediate cytokine expression [48]. Furthermore, ligand (LPS) uptake into an intracellular compartment of macrophages in a TLR4-independent mechanism is suggested to induce inflammasome complexes and enhance pro-inflammatory cytokine (IL-1β and IL-18) secretion at later time points [49]. This finding further highlights the presence of a novel and intrinsic intracellular LPS sensor in the cytoplasm of cells for initiation of NLRP3-specific inflammasome activating signal complex formation [49]. In mice and humans, the NLRP3 inflammasome (NOD-like receptor family, pyrin domain containing 3), a well characterized inflammasome, regulates caspase-1 activity that leads to the cleavage of pro-IL-1β and pro-IL-18 into their respective bioactive cytokines [50].

Although the role of inflammasomes has not been well described in chickens [51], it is plausible that LPS encapsulated into PLGA NPs may be released into the cytosol of the cells providing an intracellular source of LPS. Its detection and recognition by intracellular sensors may result in the expression of IL-1β, where surface associated LPS may have priming effects by binding to TLR4 expressed on the plasma membrane of macrophages.

The sustained systemic stimulation of spleen cells by LPS in the *in vivo* study can be attributed to particle trafficking or depot effects after parenteral injections. The trafficking of NPs, after phagocytosis by resident antigen presenting cells at the sites of injection or (recruited) inflammatory cells, to the spleen [38] may account for a sustained systemic stimulation of spleen cells. Sharp et al. (2009) have demonstrated that PLGA NPs induce local innate/inflammatory responses at the site of injection mainly via inflammasome activation [46]. Experimentally, injection of CpG ODN or *monophosphoryl lipid A* (conjugated with nanolipoproetin particulates), intraperitoneally into mice induce higher splenic expression of pro-inflammatory cytokines [38] implying parenteral administration of particulate carriers may have systemic effects. Alternatively, the depot effects of PLGA NPs may also allow the gradual release of ligands by passive diffusion or by erosion of the polymer matrix [18] with delayed and persistent cytokine responses in the spleen producing a slow release profile for LPS. An extracellular depot of PLGA particles releasing poly(I:C), a TLR3 ligand, over 4 days in lymph nodes has been shown to enhance immune responses. This allows dendritic cells and immune cells newly recruited to the lymph nodes to be continuously stimulated, mimicking sustained inflammatory signaling achieved by local infections [52].

In conclusion, the *in vitro* and *in vivo* findings presented here highlight the effectiveness of particulate delivery systems for TLR ligands to enhance activation of cells of the innate system. As one of the constituent cells of innate immunity, macrophages recognize a wide range of stimuli including TLR ligands by their surface and intracellular receptors and as such these interactions involve multiple signal transduction pathways for induction of effector molecules [1]. The innate defense capability of these cells can be fine-tuned by stimulating them with appropriate stimuli; particulate delivery that incorporates TLR ligands may enhance their antiviral and adjuvant properties [18]. Determination of appropriate doses of the ligands and delivery vehicles will require additional investigations. A priming effect, followed by a sustained stimulation of immune system cells resulting from a slow release of ligands from the polymers, may be essential to enhance antigen specific immune responses following vaccination or when TLR ligands are applied as antimicrobial compounds. Furthermore, immune-enhancing effects of TLR ligands can be improved and tailored by developing PLGA nanocarriers targeting particular tissue micro-environments. We have evaluated the TLR ligand delivery system in chicken macrophages, but not in other professional antigen presenting cells of avian origin including dendritic cells and monocytes to have broader understanding of how the delivery system can be formulated as a universal TLR ligand delivery system in chickens. A more comprehensive study of the impact of this delivery system on cells, pathways and molecules involved in host innate and adaptive responses is warranted.

## Supporting Information

S1 Table Nitric oxide production from chicken macrophages treated with encapsulated or free TLR ligands. (XLSX)

S2 Table Cytokines gene expression by chicken macrophages after treatment with encapsulated or soluble forms of TLR ligands. (XLSX)

S3 Table Live body weight gains of chickens intramuscularly injected with PLGA NPs. (XLSX)

S4 Table Cytokines gene expression in spleen of chickens intramuscularly injected with encapsulated or soluble LPS. (XLSX)
